# Anthony Montonnier Hawkins

**Published:** 1833-09

**Authors:** 


### OBITUARY.

DIED, on the 22d of July, at his house, in Upper Brook street, Grosvenor square, in his sixty-third year, ANTHONY MONTONNIER HAWKINS, Esq. M.D. By his family and a large circle of friends, who knew how to estimate his merits and the excellent qualities of his heart, his loss will be long and deeply deplored.

